# Educational technology: educational video to support home care management for liver transplant patients

**DOI:** 10.1590/0034-7167-2024-0456

**Published:** 2025-09-08

**Authors:** Neide da Silva Knihs, Evelin da Silva Della Betta, Ariadne Matzembacher da Silva, Aline Lima Pestana Magalhães, Letícia de Oliveira Grespi, Sibele Maria Schuantes Paim, João Luís Erbs Pessoa, Juliana Martins Costa

**Affiliations:** I Universidade Federal de Santa Catarina. Florianópolis, Santa Catarina, Brazil; II Universidade Federal de São Paulo. São Paulo, São Paulo, Brazil; III Secretaria da Saúde do Estado de São Paulo. São Paulo, São Paulo, Brazil

**Keywords:** Educational Technology, Liver Transplantation, Health Promotion, Postoperative Care, Treatment Adherence and Compliance, Tecnología Educacional, Trasplante Hepático, Promoción de la Salud, Cuidados Posoperatorios, Cumplimiento y Adherencia al Tratamiento

## Abstract

**Objectives:**

to develop and validate educational video to support the management of home care for clients undergoing liver transplantation.

**Methods:**

a study supported by Instructional Design, through the following stages: analysis: data obtained through three studies already developed by the researchers; design: the script learning objectives were outlined; sequences of scenes, professionals involved, location, language, illustrative figures and necessary materials. Moreover, content validity: production - video development; implementation and evaluation - the video was used by clients undergoing liver transplantation followed by their assessment of this product. Descriptive statistics were used for data analysis.

**Results:**

the video addressed five contents according to clients’ needs, being validated by six nurses with more than four years of experience in transition of liver transplant care and by nine clients with an average hospital discharge time of 31 days. The overall average score of “very good” was over 61%, followed by the “good” score, with an average of 23.8%.

**Final Considerations:**

the study enabled including professionals with expertise in the subject, with adjustments being made according to user demand. It is worth noting that this tool has the potential to support self-care in home environments.

## RESUMEN

**Objetivos:**

desarrollar y validar video educativo para apoyar la gestión de la atención domiciliaria de clientes sometidos a trasplante de hígado.

**Métodos:**

estudio apoyado en el Diseño Instruccional, a través de los pasos: análisis: datos obtenidos a través de tres estudios ya desarrollados por investigadores; design: describió los objetivos de aprendizaje del guión; secuencias de escenas, profesionales implicados, localización, lenguaje, figuras ilustrativas y materiales necesarios. Además, se llevó a cabo la validación de contenido; producción: desarrollo de videos; implementación y evaluación: el video fue utilizado por clientes sometidos a trasplante de hígado seguido de su evaluación de este producto. Se utilizó estadística descriptiva para el análisis de los datos.

**Resultados:**

el video abarcó cinco contenidos según las necesidades de los clientes, siendo validado por seis enfermeros con más de cuatro años de experiencia en la transición de la atención al trasplante hepático y por nueve clientes con un tiempo promedio de alta hospitalaria de 31 días. El promedio general de la puntuación “muy bueno” fue superior al 61%, seguido del puntaje “bueno”, con un promedio del 23,8%.

**Consideraciones Finales:**

el estudio permitió incluir profesionales con experiencia en el tema, realizando ajustes según la demanda de los usuarios. Cabe destacar que esta herramienta tiene el potencial de apoyar el autocuidado en el ámbito doméstico.

## INTRODUCTION

The complexity arising from liver transplantation indication and performance generates the need for different care strategies from the multidisciplinary team, in order to facilitate a safe and effective postoperative period, combined with adequate planning of transition of care between hospital and home, considering the particularities of each client and their family^(1, 2, 3)^.

Given this complex context, it is essential to organize each of the stages involved in the perioperative period, from patients’ inclusion on the transplant waiting list to post-discharge follow-up. Thus, teams, in partnership with patients, caregivers and families, must organize every detail of care to be provided, including post-transplant discharge care. Adapting to the new reality together with the return home requires attention to detail and care management from teams, patients and families. All of these factors impact complication prevention, treatment compliance and graft survival^(4, 5)^.

Failure to comply with care and medication use can lead to possible complications, such as graft loss and even death. The appearance of these complications and/or complications can trigger negative feelings, such as anxiety and insecurity^(6, 7)^. In this regard, it is necessary to develop a care transition plan between hospital and home in an efficient and comprehensive manner, supported by multidisciplinary team, client and support network engagement^(8)^.

Considering the reality exposed, health education emerges as a strategy that impacts the improvement of clients and family members, especially regarding the understanding and development of skills to conduct home care techniques. Continuity of care after hospital discharge is long and continuous. Thus, clients must be able to promote self-care with the support of a family member or caregiver and, above all, be able to identify any sign or symptom of possible complications and complications as soon as possible^(1, 6, 7)^.

Given this scenario and the complexity of the liver transplant procedure, along with home care, technologies are resources that favor the process of teaching, learning and skill development. Technologies that use audio and video resources have the advantage of optimizing communication with the multidisciplinary team, since they are accessible to clients’ realities and arouse interest in learning^(6, 9, 10)^.

Among the technologies already used to support health education, educational videos stand out. Some initiatives in this sense have already been used, such as videos produced to support care for clients with stomas, videos for necessary guidance in the post-operative period of mastectomy and content development and validity for educational videos on dietary adjustments for hypertensive clients^(11, 12, 13)^. The authors highlight that video-based learning is light and subtle, reinforcing the public’s attention to promoting self-care^(11)^.

Furthermore, videos allow clients to observe the procedures performed in practice at any time, with the flexibility to pause and review several times, respecting each person’s learning time, thus contributing to health promotion, as it enables and encourages greater autonomy for these people^(13, 14, 15)^.

Given the development of educational videos as a health promotion strategy in different areas of care, the opportunity to develop this type of technology for clients in the context of liver transplantation was observed. Considering the level of detail and complexity of care, the strategy can be useful for this population, especially in transition of care between hospital and home, in order to provide self-confidence and improve the conduct of care delivery.

It is worth noting that educational videos for healthcare present the challenge of being developed based on evidence-based information and presented in a way that is accessible to users. Therefore, the effective participation of users in the development and validity stages favors the development of technology in a way that is centered on individuals’ needs and understanding, with language adjustments and audiovisual resources specific to them^(13, 16, 17)^.

In this regard, the study raises the following guiding question: what information should be included in an educational video to promote health and support transition of care between hospital and home for clients undergoing liver transplantation?

## OBJECTIVES

To develop and validate an educational video to support home care management for clients undergoing liver transplantation.

## METHODS

### Ethical aspects

The study was conducted in accordance with national and international ethics guidelines, and was approved by the *Universidade Federalde Santa Catarina* Research Ethics Committee.

### Study design, period and place

This is a technological production study, which is anchored by the Analysis, Design, Development, Implementation and Evaluation (ADDIE) method^(18)^ stages: 1) analysis; 2) design; 3) development and production; 4) implementation; and 5) evaluation. The study is part of a macroproject entitled “*Transplante hepático em Santa Catarina: caracterização egerência do cuidado para a melhoria do processo”.*

The study stages were developed in 2023 and 2024 at a large public hospital institution in the south of the country, which is a reference in liver transplantation.

### Population, sample, inclusion and exclusion criteria

The study included patients who underwent liver transplantation between 2011 and 2022 at the aforementioned hospital as well as their family members/caregivers. The sample was random, non-probabilistic and intentional, since it sought to include in the sample patients at different times after liver transplantation who were already discharged from hospital. These patients should have been discharged up to 12 months after transplant and be over 18 years old. Those who only formalized outpatient care at the institution were not included, as they were not introduced to the care and guidance to be performed in transition to home care at that institution. The sample was random, intentional and non-probabilistic.

Moreover, nurses with specific expertise in liver transplantation focused on transition of care between hospital and home and who had been working in this context for more than two years were included. This criterion was defined because professionals with experience and experience can more sensitively assess the video content and applicability. The sample was random, non-probabilistic and intentional, because the authors were concerned about including professionals with different lengths of experience in transition of care in the study. To identify these professionals, reference hospitals for liver transplantation were contacted, identified through the Brazilian Association of Organ Transplantation.

### Methodological procedures

Data collection will be presented through each stage of the ADDIE method.

1^st^ stage: analysis – for this stage, three studies already developed during the execution of the macroproject of which the study is a part were used. The first survey identified home care needs for post-liver transplant patients that were reported in scientific literature^(19)^. The second study explored experiences of patients during post-liver transplantation at home using a qualitative approach^(1)^. Finally, the third study, which supported the first stage of the educational vide development, was the creation of a mobile application for health education^(20)^.

Information from the three previous studies supported content development to compose the educational video. However, to conduct this stage, three meetings were held between the research members, composed of professors, undergraduate and graduate students. After the research members had previously read the materials, the first meeting was scheduled, in which the researchers presented the priority care already mapped by studies.

At the end of the first meeting, the researchers reached a consensus on the information that should make up the video based on the recording units. The second meeting aimed to script the video, based on the document developed in the previous meeting. After the document was presented, the project team discussed the content topics that would make up the video script.

The last meeting was for reading and rereading the sequence of contents as a way of consolidating the content discussed in the previous meetings. Moreover, the objective that each scene would have from the perspective of care was discussed. The average length of the meetings was 45 minutes, and all were recorded and transcribed.

2^nd^ stage: design – in this stage, expectations were defined regarding the video script learning objectives and the learning to be acquired by clients through viewing this educational tool. For this purpose, a new meeting was held with the project team.

At this meeting, the script for the video was defined based on learning objectives, materials, characters and location where each of the stages of the video would be produced, including the sequences of scenes, professionals involved, filming locations, language, illustrative figures and necessary materials. After the script was written, a new meeting was held to discuss adjustments with the researchers and finalize the script.

With the script finalized, it was sent for content validity, i.e., at this point in the study, there was the first data collection regarding video content validity by nursing professionals who work in the context of transition of care for liver transplant clients.

To collect data, the researchers initially contacted the institution by email to present the research. After authorization from the institution, contact was made with the professionals. Following the affirmative response to participate in the research, the Informed Consent Form (ICF) was sent to participants.

After signing the ICF, participants also received an online Google form link via email or WhatsApp®, which included the video script and the data collection questionnaire regarding content validity. The questionnaire followed the content validity parameters based on criteria of objectivity, simplicity, clarity, credibility, relevance and agreement^(21)^. Each item presented the possible responses on a Likert scale, ranging from disagree, partially disagree, partially agree and agree.

For each participant, the response to the online form was requested to be sent within 30 days, and, after sending it, professionals were invited to recommend three more nursing colleagues with the same work experience for the study, understanding the snowball technique for recruiting participants.

3^rd^ stage: development and production – this stage involves the production of the video itself, which begins after the script has been validated by professionals. Initially, the space in the Skills Laboratory at the institution where the main researcher works as a professor was reserved. This laboratory has different types of simulators and other equipment and materials for health practices. The recording was developed by project participants using cameras and tripods from the laboratory itself. One of the project nurses directed the scenes, and the other members worked on the organization and as actors. Filming took approximately seven hours. Then, the separately recorded scenes were edited using the CANVA platform, composing two videos with an average duration of 20 minutes.

4^th^ and 5^th^ stages: implementation and evaluation – the implementation stage refers to the movement of inserting the developed video into the target audience’s reality, i.e., post-liver transplant clients. To this end, it was initially chosen to implement it with clients who had already been discharged from hospital after transplantation, since they already had experience with transition of care.

Along with this stage, video assessment was developed through the second data collection. Data collection began with the researchers contacting the clients who are being monitored at the institution’s transplant clinic.

Those clients who showed interest in participating in the survey received the ICF by email or WhatsApp®. Upon accepting to participate in the survey, participants had access to the link to the developed video made available on YouTube® and a link to an online Google form to continue the evaluation.

The video assessment form presented the following questions: video length; figures, characters and materials in the video; contributes to improving knowledge about home care; information in the video and the step-by-step guide to performing care are clear; and information can improve care and support the management of this care at home. For each of these questions, participants responded using a Likert scale, between poor, reasonable, good or very good. In addition to this, at the end of the form, there was a space for comments and suggestions for changes in the video content, scenes and length. A maximum time limit of 30 days was set for participants to submit their responses.

### Data analysis

The data were organized in a Microsoft Excel® spreadsheet, and content validity analysis by nursing professionals and video assessment by clients were carried out using descriptive statistics, based on measures of central tendency and absolute frequencies (n) and relative frequencies (%).

## RESULTS

The results will be presented according to the stages of the ADDIE method already described, in order to clarify the findings.

In the first stage, analysis, the following recording units were defined as topics to compose the video: 1) home restrictions; 2) oral hygiene; 3) home environment cleaning; 4) blood glucose monitoring; 5) essential care of surgical wound; 6) insulin application; 7) daily controls. Then, after discussion with the project team, the following were defined to compose the video contents: 1) environment organization and cleaning; 2) body care; 3) blood glucose monitoring; 4) insulin application; 5) development of daily controls and warning signs for intercurrences and complications.

In the design phase, the video script was detailed based on the learning objectives. The video script is made up of the five topics mentioned above with details of scenes, materials, lines, physical space and other relevant information.

The full script is available as supplementary material at the following link: https://doi.org/10.17605/OSF.IO/JCFMP. Chart 1 presents an example of one of the scenes from the script, focusing on blood glucose development and monitoring. This chart aims to elucidate the script development process. The first column presents the learning objective, while the second column provides a description of one of the scenes from the script.

**Chart 1:** Learning objective of checking blood glucose/script excerpt from this stage of the video, Florianópolis, Santa Catarina, Brazil, 2024

Learning objective	Example of a script description for one of the scenes/stages in the video
Support clients, family and caregivers in developing and monitoring blood glucose levels.	Hello, welcome, this is a video to support the client, family and caregiver in managing home care. Here, we will guide and assist in the step-by-step process of monitoring blood glucose, an essential care for the continuity of home treatment. We will begin by presenting the materials needed to perform the blood glucose test. The first step is to ensure that you have all the materials: glucometer - a device to measure the level of glucose (popularly known as “sugar”) in the blood; lancet; test strip; cotton; and 70% liquid alcohol. These materials will be provided by the health team at the time of your discharge from the hospital through your Basic Health Unit. Do not worry, you won’t need to buy anything. It is important to note that this blood glucose test must be performed for some time, also at home. It is necessary because the organ is recovering, due to the use of high doses of immunosuppressants and corticosteroids. As time passes and the organ recovers, in addition to adjustments in the medication, this test will no longer be necessary. The healthcare team that is monitoring you will inform you when there is no longer a need to monitor your glucose levels. Attention: show how to clean the workbench, talk about the importance of cleaning, and then show and point out the materials on the workbench.

**Figure 1:**
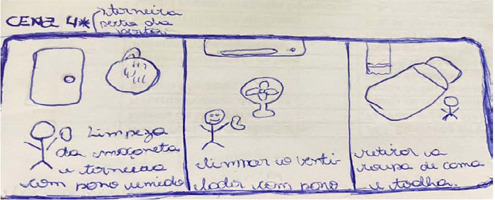
Example of a scenario from one of the stages of the video “Home care related to hygiene at home”, Florianópolis, Santa Catarina, Brazil, 2024

Along with the description of scenes, the researchers created the scene ambience through simple, hand-drawn drawings in order to demonstrate the scenarios in order to better approximate the reality experienced in clients’ homes. Figure 1 shows an example of the explanation of scenes through simple drawings.

The script content validity was performed by six nurses who work in care practice, with an average age of 44.5 years. Among participants, 28.6% have four or more years of experience in transition of care/hospital discharge of liver transplantation; 42.9% have a master’s degree; 51.1% work in a public institution; and 57.1% currently perform the care function with the multidisciplinary liver transplant team.

Table 1 presents the main data related to content validity in terms of agreement among experts on the criteria. It is noted that the overall average for all criteria was higher than 83%. The criteria with the highest agreement were credibility, with a percentage above 89%, followed by the agreement criterion with 84.68%. It is also worth noting that disagreement and partial disagreement scores were not recorded.

**Table 1:** Experts’ assessment (N=06) regarding the content validity of the video scenes, Florianópolis, Santa Catarina, Brazil, 2024

Criteria	Agree (%)	Partially agree (%)	Partially disagree (%)	Disagree (%)
Objectivity	82.13	17.87	00	00
Simplicity	80.18	19.82	00	00
Clarity	82.96	17.04	00	00
Credibility	89.72	15.27	00	00
Relevance	84.17	15.85	00	00
Agreement	84.68	15.32	00	00
Mean	83.97	16.81	00	00

Chart 2 presents below an example of the script adjusted by the researchers after adjustments to recommendations by evaluators. The chart shows the importance of consulting experts, professionals in practice, because it is possible to observe that there were important adjustments and fundamental additions of information.

**Chart 2:** Demonstration of the script prepared by the researchers and after suggestions for adjustments by experts, Florianópolis, Santa Catarina, Brazil, 2024

Script excerpt defined by the researchers	Script excerpt after adjustments to suggestions by experts
Regarding the non-use of animals, it is important to note that they contain bacteria, viruses and other microorganisms that are invisible to our eyes. However, any contact with the client, who already has low immunity, can result in the acquisition of these bacteria, viruses and other contaminations that may be present in animals.	Regarding the non-use of animals, it is important to minimize or avoid contact with animals, as well as not handling your pet’s feces and urine. It is very important, at this stage, that you avoid sleeping with your pet and keep all vaccinations up to date to prevent diseases. Animals have bacteria, viruses and other microorganisms that are invisible to our eyes; therefore, it is necessary to wash your hands after playing. Any contact with the client, who already has low immunity, can result in the acquisition of these bacteria, viruses and other contaminations, which may be present in animals.

After the script content was validated by nurses, the third stage was conducted in relation to video development and production. The video was produced, edited and made available on YouTube®, and can be accessed through the links: https://youtu.be/jQwPgwY54ZY and https://youtu.be/qaI9RxtEIko.

For the last two stages, implementation and evaluation, nine clients participated in the research, five men and four women. The average age was 44.5 years, and the average time of transition of care between hospital and home was 31 days.

Table 2 shows the results regarding video assessment by clients. The overall average score of “very good” was over 61%, followed by the “good” score, with an average of 23.8%, which indicates a positive assessment of the technology developed. The criteria best assessed by the “very good” score were in relation to the video’s contribution to knowledge of home care, information on stages of care and perspective of this information in improving home care.

**Table 2:** Video assessment by participants (N = 09 clients), Florianópolis, Santa Catarina, Brazil, 2024

Criteria	Very good (%)	Good (%)	Reasonable (%)	Poor (%)
1 – Video length	42.8	42.8	14.4	00
2 – Figures, characters and materials in the video	50.0	33.3	16.7	
3 – Contributes to improving knowledge about home care	71.4	14.3	00	14.3
4 – Information in the video and the step-by-step guide to providing care are clear	71.4	14.3	00	14.3
5 – Information can improve care and support the management of home care	71.4	14.3	00	14.3
Mean	61.4	23.8	6.2	8.6

## DISCUSSION

Health education, through technology, represents a valuable strategy to help with post-operative difficulties, given that educational videos serve as improvement tools for family members, caregivers and patients undergoing liver transplantation. Studies indicate that patients often have difficulties and weaknesses in performing the necessary techniques and/or care that must be performed at home. Furthermore, there are concerns about the care and maintenance of a home environment that is suitable for patients’ reintegration upon returning home after transplant. Thus, it is important for the multidisciplinary team to have effective strategies to minimize these insecurities or doubts that may arise after discharge, considering that failure to understand these precautions can lead to problems related to ineffective control, poor management of care and inadequate hygiene. Such situations increase the risk of complications^(22, 23)^.

In this study, the researchers proposed to identify the care needs of these clients, by developing a video script to meet their individual needs and provide essential information about home care. It is important to highlight the need to develop tools that are able to serve users. Only then will these tools be used and be able to achieve the desired effectiveness for the proposed care objectives^(24, 25, 26)^.

During the study development, the researchers focused their attention on improving the information in the script with the support of nurses who work in transition of care. This stage is considered important, since these professionals can assess and adjust home care management strategies based on the daily care provided.

Studies highlight the importance of the product being assessed by professionals with experience in the field, with the purpose of adding practical input to the tool to be developed, providing suggestions and modifications so that the product or technology is accessible to the target audience. Assessment is crucial for several reasons, such as: validating the video quality, ensuring that it meets standards and recommended practices; identifying problems or weaknesses; and offering alternatives. In addition, feedback from experts helps with the video refinement, technical feasibility and improvement^(27, 28, 29)^.

Furthermore, as pointed out by different authors, it is important to include users in all stages of the process of creating educational materials, since this inclusion allows the tool to be simple and accessible, and easy for the target audience to understand and manipulate. This approach to reality includes the daily situation of each client, favoring the availability of space to clarify doubts as well as understanding that such information is capable of empowering and supporting the conduct of home care^(24, 28, 30)^.

In this study, the video was made available to clients, family members and caregivers who were in the process of adapting to their homes, which represent moments that require home care and monitoring. Thus, they had the possibility of accessing this tool whenever they deemed necessary. Studies indicate that, considering the diversity of users who use care tools, these need to be used for a different period, seeking to assess the understanding and acceptance of this product^(31, 32)^.

It is worth noting that the tool for this study was made available via YouTubeº as a quick and easy search resource, as recommended in the development of methods for preparing and creating educational materials. It is understood that Information and Communication Technologies in Health (ICTHs) allow the storage and processing of digital data, access to information and remote communications. Therefore, the use of ICTHs seeks to assist and optimize assistance for decision-making both in clinical practice and in monitoring, assessing actions and Continuing Education in Health^(33)^.

From the perspective of user use and assessment, the participation of this audience allows the tool to be improved, making it educational for future users. By defining feasible goals and objectives for the video function, users used the videos for a month, making it possible to identify the need for changes. Authors point out that user participation enriches the context of the tool, in addition to being as close as possible to the demand, with reformulations and adjustments that make the product more accessible^(29, 34)^.

Other studies that produced videos with user participation had similar results to this study. The authors emphasize that this educational video tool allows clients to observe the procedures performed in practice at any time, with the flexibility to pause and review them several times, making it an effective health promotion strategy^(14, 15)^. However, such technologies must be developed with the effective participation of users at all stages, since, to have a positive impact, they must be evaluated and adjusted by people who effectively have experience in the reality of the change in routine imposed by transplant, bringing them closer to the lived context and encouraging representation in the development of care strategies^(16, 17, 35)^.

Since they are illustrative and interactive tools, educational videos are well accepted by the public in terms of usability and dissemination. They also encourage the opportunity for users to improve their knowledge after using the video^(25, 36)^, generating an impact on quality of life of transplant recipients.

Considering such data presented, as well as the results obtained in other studies cited above, it is clear that the application of the tool from this study in daily guidance for hospital discharge can bring important benefits, both for clients who will enjoy this video whenever they wish, and for the multidisciplinary team, which will have this additional care strategy as an instrument in adhering to treatment for clients undergoing liver transplantation.

### Study limitations

The limitations of this study are related to difficulties in identifying nursing professionals with expertise in transition of care between hospital and home with a focus on liver transplantation. Furthermore, the educational video was validated by clients who underwent liver transplantation in a single institution.

### Contributions to nursing

This study provides an improvement in the management of home care because clients who underwent a liver transplant, together with their family member and/or caregiver, can access information related to home care at any time. Thus, there is the possibility of strengthening health education and care management, in addition to supporting the multidisciplinary transplant team through an educational tool, improving treatment adherence and providing autonomy to clients. The results of the validity between expert professionals and clients demonstrate the feasibility of including the educational tool in the routine of preparing for hospital discharge of liver transplant clients, contributing to transition between hospital and home.

## FINAL CONSIDERATIONS

The study developed and validated a video to promote health education for clients undergoing liver transplantation. The aforementioned educational tool was formed by scenes in which the step-by-step daily care to be carried out at home is presented, focused on: oral hygiene; blood glucose monitoring; surgical wound care; insulin application; daily controls (checking vital signs; measuring diuresis and weight); and development of daily controls and warning signs for incidents and complications. It also includes scenes related to home care and personal hygiene, such as environment organization and cleaning, care with the use of masks, and home visits.

Furthermore, it is worth noting that this video was implemented with clients undergoing liver transplantation, assessing whether this tool meets their needs at home.

Agreement between professional expert and client assessments demonstrates that the assessments were considered good, which indicates that the educational video has validated its content. Furthermore, this tool can be used by clients as well as by the multidisciplinary team in the transition of care between the hospital and the home of liver transplant clients.

## Data Availability

https://doi.org/10.17605/OSF.IO/JCFMP
