# Supplementary material for: Educational technology: educational video to support home care management for liver transplant patients
Source: Rev Bras Enferm. 2025 Sep 8;78(3):e20240456. doi: 10.1590/0034-7167-2024-0456 (PMC12419785; doi:10.1590/0034-7167-2024-0456)
Supplement: Translation

# Translation: Tecnologia educacional: vídeo educativo para apoiar a gestão do cuidado domiciliar para clientes submetidos ao transplante hepático

**DOI:** 10.1590/0034-7167-2024-0456pt

**Authors:** Neide da Silva Knihs, Evelin da Silva Della Betta, Ariadne Matzembacher da Silva, Aline Lima Pestana Magalhães, Letícia de Oliveira Grespi, Sibele Maria Schuantes Paim, João Luís Erbs Pessoa, Juliana Martins Costa

**Affiliations:** I Universidade Federal de Santa Catarina. Florianópolis, Santa Catarina, Brasil; II Universidade Federal de São Paulo. São Paulo, São Paulo, Brasil; III Secretaria da Saúde do Estado de São Paulo. São Paulo, São Paulo, Brasil

**Keywords:** Tecnologia Educacional, Transplante Hepático, Promoção da Saúde, Cuidados Pós-Operatórios, Cooperação e Adesão ao Tratamento

## Abstract

**Objetivos:**

desenvolver e validar vídeo educativo para apoiar a gestão do cuidado domiciliar para clientes submetidos ao transplante hepático.

**Métodos:**

estudo sustentado pelo *Design* Instrucional, por meio das etapas: análise: dados obtidos por meio de três estudos já desenvolvidos pelos pesquisadores; *design:* traçados os objetivos de aprendizagem do roteiro; sequências das cenas, profissionais envolvidos, local, linguagem, figuras ilustrativas e materiais necessários. Ainda, ocorreu a validação de conteúdo; produção: desenvolvimento do vídeo; implementação e avaliação: o vídeo foi utilizado pelos clientes submetidos ao transplante hepático seguido de avaliação deste produto por eles. Utilizou-se para análise dos dados estatística descritiva.

**Resultados:**

o vídeo abordou cinco conteúdos de acordo com as necessidades dos clientes, sendo validado por seis enfermeiros com mais de quatro anos de experiência na transição do cuidado do transplante hepático e por nove clientes com tempo médio de alta hospitalar de 31 dias. A média geral do escore “muito bom” foi superior a 61%, seguido do escore “bom” com uma média de 23,8%.

**Considerações Finais:**

estudo possibilitou a inserção de profissionais com *expertise* na temática, com a realização de ajustes de acordo com a demanda dos usuários. Destaca-se que essa ferramenta tem potencial para apoiar o autocuidado em ambiente domiciliar.

## INTRODUÇÃO

A complexidade advinda da indicação e realização do transplante hepático gera a necessidade de diferentes estratégias de cuidados da equipe multiprofissional, no sentido de viabilizar um pós-operatório seguro e efetivo, aliado a planejamento adequado da transição do cuidado entre hospital e domicílio, considerando as particularidades de cada cliente e de sua família^(1, 2, 3)^.

Diante desse contexto complexo, torna-se fundamental a organização de cada uma das etapas que envolvem o perioperatório, desde a inserção do cliente em lista de espera para o transplante até o acompanhamento pós-alta. Assim, as equipes, em parceria com cliente, cuidador e família, devem organizar cada detalhe do cuidado a ser realizado, incluindo os cuidados da alta hospitalar no pós-transplante. A adaptação à nova realidade junto com o retorno ao domicílio exige da equipe, cliente e familiares atenção aos detalhes e gestão do cuidado. Todos esses fatores impactam a prevenção de agravos, adesão ao tratamento e sobrevida do enxerto^(4, 5)^.

A não adesão aos cuidados, bem como ao uso de medicamentos, leva a possíveis intercorrências e complicações, como a perda do enxerto e até mesmo óbito. O aparecimento dessas intercorrências e/ou complicações pode despertar sentimentos negativos, como ansiedade e insegurança^(6, 7)^. Nesse sentido, é necessário que seja desenvolvido um planejamento de transição do cuidado entre hospital e domicílio de forma eficiente e integral, sustentado pelo engajamento da equipe multiprofissional, bem como dos clientes e rede de apoio^(8)^.

Considerando a realidade exposta, a educação em saúde surge como estratégia que impacta o aprimoramento do cliente e familiares, especialmente quanto à compreensão e desenvolvimento de habilidades para conduzir as técnicas dos cuidados em domicílio. A continuidade do cuidado após a alta hospitalar é longa e contínua. Assim, o cliente deve estar apto a promover seu autocuidado com apoio de um familiar ou cuidador e, acima de tudo, ser capaz de identificar qualquer sinal ou sintoma de possível intercorrência e complicação o mais breve possível^(1, 6, 7)^.

Diante deste cenário e da complexidade do procedimento do transplante hepático, junto aos cuidados domiciliares, as tecnologias são recursos que favorecem o processo de ensino, aprendizagem e desenvolvimento de habilidades. Tecnologias que utilizam recursos de áudio e vídeo apresentam a vantagem de otimizar a comunicação com a equipe multiprofissional, uma vez que é acessível às realidades dos clientes e despertam interesse pelo aprendizado^(6, 9, 10)^.

Entre as tecnologias já utilizadas para apoio à educação em saúde, destacam-se os vídeos educativos. Algumas iniciativas nesse sentido já vêm sendo utilizadas, a exemplo de vídeos produzidos, a fim de apoiar os cuidados a clientes com uso de estomas, vídeos para orientações necessárias no pós-operatório de mastectomia e desenvolvimento e validação de conteúdo para vídeo educativo sobre ajustes de dieta para clientes hipertensos^(11, 12, 13)^. Os autores destacam que o aprendizado por vídeo é leve e sutil, reforçando a atenção do público para a promoção do autocuidado^(11)^.

Ademais, vídeos permitem que os clientes observem na prática os procedimentos realizados a qualquer momento, com a flexibilidade de pausar e rever diversas vezes, respeitando o tempo de aprendizado de cada um, contribuindo, assim, para promoção da saúde, uma vez que possibilita e incentiva maior autonomia para essas pessoas^(13, 14, 15)^.

Diante do desenvolvimento de vídeos educativos como estratégia de promoção à saúde em diferentes áreas do cuidado, observou-se a oportunidade de desenvolvimento deste tipo de tecnologia para os clientes inseridos no contexto do transplante hepático. Considerando o nível de detalhe e complexidade do cuidado, a estratégia pode ser útil para esta população, especialmente na transição do cuidado entre hospital e domicílio, no sentido de proporcionar autoconfiança e melhora na condução da realização do cuidado.

Vale destacar que vídeos educativos para o cuidado em saúde apresentam o desafio de serem desenvolvidos a partir de informações baseadas em evidências e apresentados de maneira acessível para os usuários. Portanto, a participação efetiva dos usuários nas etapas de desenvolvimento e validação favorece o desenvolvimento da tecnologia de maneira centrada nas necessidades e na compreensão do indivíduo, com ajustes de linguagem e recursos audiovisuais específicos para eles^(13, 16, 17)^.

Neste sentido, o estudo traz como questão norteadora: quais informações devem compor um vídeo educativo para promoção da saúde e apoio à transição do cuidado entre hospital e domicílio para clientes submetidos ao transplante hepático?

## OBJETIVOS

Desenvolver e validar vídeo educativo para apoiar a gestão do cuidado domiciliar para clientes submetidos ao transplante hepático.

## MÉTODOS

### Aspectos éticos

O estudo foi conduzido de acordo com as diretrizes de ética nacionais e internacionais, sendo aprovado pelo Comitê de Ética em Pesquisa da Universidade Federal de Santa Catarina.

### Desenho, período e local do estudo

Estudo de produção tecnológica, o qual está ancorado pelas etapas do método *Analysis, Design, Development, Implementation* e *Evaluation* ^(18)^ (ADDIE): 1) análise; 2) *design;* 3) desenvolvimento e produção; 4) implementação; e 5) avaliação. O estudo faz parte de macroprojeto intitulado “Transplante hepático em Santa Catarina: caracterização e gerência do cuidado para a melhoria do processo”.

Desenvolveram-se as etapas do estudo no período de 2023 e 2024 em instituição hospitalar pública, de grande porte no sul do país, que é referência no transplante hepático.

### População, amostra, critérios de inclusão e exclusão

Participaram do estudo os clientes que foram submetidos ao transplante hepático, entre 2011 e 2022, no referido hospital, assim como seus familiares/cuidadores. A amostra foi aleatória, não probabilística e intencional, haja vista que buscou incluir na amostra clientes em diferentes tempos de pós-transplante hepático que já estavam em alta hospitalar. Esses deveriam ter até 12 meses de alta hospitalar após o transplante e ser maiores de 18 anos. Não foram incluídos aqueles que formalizaram apenas o atendimento ambulatorial na instituição, pois não foram introduzidos aos cuidados e orientações para serem desempenhados na transição para domicílio na referida instituição. A amostra foi aleatória, intencional e não probabilística.

Além desses, foram incluídos os enfermeiros com *expertise* específica na temática do transplante hepático direcionada à transição do cuidado entre hospital e domicílio e que atuavam nesse contexto há mais de dois anos. Esse critério foi definido porque profissionais com experiência e vivência podem avaliar de maneira mais sensível o conteúdo e aplicabilidade do vídeo. A amostra foi aleatória, não probabilística e intencional, em razão dos autores terem a preocupação de inserir no estudo profissionais com diferente tempo de atuação na transição do cuidado. Para identificação desses profissionais, foi feito contato com hospitais de referência em transplante hepático, identificados por meio da Associação Brasileira de Transplante de Órgãos.

### Procedimentos metodológicos

A coleta dos dados será apresentada por meio de cada etapa do método ADDIE.

1ª etapa: análise – para esta etapa, foram utilizados três estudos já desenvolvidos durante a execução do macroprojeto do qual o estudo faz parte. A primeira pesquisa identificou as necessidades de cuidado em domicílio para os clientes do pós-transplante hepático que foram relatadas na literatura científica^(19)^. A segunda pesquisa explorou a vivência dos clientes durante o pós-transplante hepático em domicílio a partir da abordagem qualitativa^(1)^. Por fim, o terceiro estudo, que fundamentou a primeira etapa do desenvolvimento do vídeo educativo, foi o de criação de aplicativo móvel para educação em saúde^(20)^.

As informações dos três estudos anteriores fundamentaram o desenvolvimento do conteúdo para compor o vídeo educacional. Contudo, para a condução desta etapa, foram realizadas três reuniões entre os membros da pesquisa, compostos por docentes, estudantes de graduação e pós-graduação. Após leitura prévia dos materiais pelos membros da pesquisa, foi agendada a primeira reunião, em que os pesquisadores apresentaram os cuidados prioritários já mapeados pelos estudos.

Ao final da primeira reunião, os pesquisadores entraram em consenso sobre as informações que deveriam compor o vídeo a partir de unidades de registros. A segunda reunião teve como objetivo roteirizar o vídeo, a partir do documento desenvolvido na reunião anterior. Após a apresentação do documento, houve discussão pela equipe do projeto, sendo definidos os tópicos de conteúdo para compor o roteiro do vídeo.

A última reunião foi para leitura e releitura da sequência dos conteúdos como maneira de consolidar o conteúdo discutido nas reuniões anteriores. Ademais, discutiu-se o objetivo que cada cena teria na perspectiva do cuidado. O tempo médio das reuniões foi de 45 minutos, e todas foram gravadas e transcritas.

2a etapa: *design* – nesta etapa, foram definidas as expectativas em relação aos objetivos de aprendizagem do roteiro do vídeo e ao aprendizado a ser adquirido pelos clientes por meio da visualização desta ferramenta educacional. Para isso, ocorreu uma nova reunião com a equipe do projeto.

Nessa reunião, foi definido o roteiro do vídeo a partir dos objetivos de aprendizagem, materiais, personagens e o local onde seria produzida cada uma das etapas do vídeo, incluindo as sequências das cenas, profissionais envolvidos, locais de filmagem, linguagem, figuras ilustrativas e materiais necessários. Após o término da escrita do roteiro, realizou-se nova reunião para discussão dos ajustes com os pesquisadores e finalização do roteiro.

Com a finalização do roteiro, este foi enviado para validação do conteúdo, ou seja, neste momento do estudo, houve a primeira coleta de dados referente à validação do conteúdo do vídeo por profissionais enfermeiros que trabalham no contexto da transição do cuidado de clientes transplantados hepáticos.

Para a coleta dos dados, inicialmente, os pesquisadores fizeram contato com a instituição por *e-mail* apresentando a pesquisa. Após autorização da instituição, o contato foi feito com os profissionais. Na sequência da resposta afirmativa em participar da pesquisa, foi enviado ao participante o Termo de Consentimento Livre e Esclarecido (TCLE).

Após assinatura do TCLE, o participante recebeu também por *e-mail* ou *WhatsApp*® um *link* de formulário *online* do *Google* que constavam o roteiro do vídeo e o questionário de coleta de dados referente à validação do conteúdo. O questionário seguiu os parâmetros de validação de conteúdo a partir de critérios de objetividade, simplicidade, clareza, credibilidade, pertinência e concordância^(21)^. Cada item apresentou as possibilidades de respostas em escala Likert, variando entre discordo, discordo parcialmente, concordo parcialmente e concordo.

Para cada participante, foi solicitado que a resposta ao formulário *online* fosse enviada em até 30 dias, e, após o envio, o profissional era convidado a indicar para o estudo mais três colegas enfermeiros com a mesma experiência de trabalho, compreendendo a técnica *snowball* para recrutamento de participantes.

3ª etapa: desenvolvimento e produção – esta etapa compreende a produção do vídeo em si, iniciada após a validação do roteiro pelos profissionais. Inicialmente, foi reservado o espaço do Laboratório de Habilidades na instituição onde a pesquisadora principal atua como docente. Neste laboratório, há diferentes tipos de simuladores e demais equipamentos e materiais para práticas de saúde. A gravação foi desenvolvida pelos participantes do projeto por meio de cameras e tripés do próprio laboratório. Uma das enfermeiras do projeto conduziu as cenas, e os demais membros atuaram na organização e como atores. O tempo para as filmagens foi de, aproximadamente, sete horas. Em seguida, as cenas gravadas separadamente foram editadas pela plataforma CANVA, compondo dois vídeos com tempo médio de 20 minutos de duração.

4ª e 5ª etapas: implementação e avaliação – a etapa de implementação se refere ao movimento de inserir o vídeo desenvolvido na realidade do público-alvo, ou seja, dos clientes após transplante hepático. Para isso, escolheu-se, inicialmente, implementar aos clientes que já haviam recebido alta hospitalar após o transplante, uma vez que já tinham a experiência da transição do cuidado.

Juntamente com essa etapa, desenvolveu-se a avaliação do vídeo, por meio da segunda coleta de dados. A coleta teve início com o contato feito pelos pesquisadores aos clientes que fazem acompanhamento no ambulatório de transplante da instituição.

Aqueles clientes que demonstraram interesse em participar da pesquisa receberam o TCLE por *e-mail* ou *WhatsApp*®. Ao aceitar participar da pesquisa, os participantes tiveram acesso ao *link* do vídeo desenvolvido disponibilizado no *YouTube*® e um *link* de formulário *online* do *Google* para prosseguir com a avaliação.

O formulário de avaliação do vídeo apresentava as seguintes questões: duração do vídeo; figuras, personagens e materiais do vídeo; contribui para o aprimoramento do conhecimento quanto aos cuidados domiciliares; informações do vídeo e o passo a passo para realizar o cuidado estão claros; e informações podem melhorar os cuidados e apoiar a gestão desses cuidados em domicílio. Para cada dessas questões, os participantes responderam, por meio de escala de Likert, entre ruim, razoável, bom ou muito bom. Além disso, ao final do formulário, havia um espaço para comentários e sugestões de mudanças no conteúdo, cenas e duração do vídeo. Foi determinado tempo máximo de envio das respostas pelos participantes de 30 dias.

### Análise dos dados

Os dados foram organizados em uma planilha do *Microsoft Excel*®, e a análise da validação do conteúdo pelos profissionais enfermeiros e a avaliação do vídeo pelos clientes foram realizadas por meio de estatística descritiva, a partir das medidas de tendência central e das frequências absolutas (n) e frequências relativas (%).

## RESULTADOS

Os resultados serão apresentados conforme as etapas do método ADDIE já descritas, no sentido de clarificar os achados.

Na primeira etapa, análise, foram definidas as seguintes unidades de registro como tópicos para compor o vídeo: 1) restrições em domicílio; 2) higiene oral; 3) limpeza do ambiente domiciliar; 4) monitoramento da glicemia; 5) cuidados essenciais com a ferida operatória; 6) aplicação da insulina; 7) controles diários. Em seguida, após discussão da equipe do projeto, foram definidos para compor os conteúdos do vídeo: 1) organização e limpeza do ambiente; 2) cuidados com o corpo; 3) monitoramento da glicemia; 4) aplicação de insulina; 5) desenvolvimento dos controles diários e sinais de alerta para intercorrências e complicações.

Na fase de *design,* o roteiro do vídeo foi detalhado a partir dos objetivos de aprendizagem. O roteiro do vídeo está formado pelos cinco tópicos supracitados com o detalhamento das cenas, materiais, falas, espaço físico e outras informações pertinentes.

O roteiro completo está disponível como material adicional conforme link: https://doi.org/10.17605/OSF.IO/JCFMP. No Quadro 1, é apresentado um exemplo de uma das cenas do roteiro, desenvolvimento e monitoramento da glicemia. Esse quadro traz a proposta de elucidar o processo de criação do roteiro. Na primeira coluna o objetivo de aprendizagem e na segunda coluna a descrição de uma das cenas do roteiro.

**Quadro 1:** Objetivo de aprendizagem da verificação da glicemia/recorte do roteiro desta etapa do vídeo, Florianópolis, Santa Catarina, Brasil, 2024

Objetivo de aprendizagem	Exemplo da descrição de roteiro de uma das cenas/etapas do vídeo
Apoiar o cliente, família e cuidador no desenvolvimento e monitoramento da glicemia.	Olá, seja bem-vindo, esse é um vídeo para apoiar o cliente, família e cuidador na gestão do cuidado em domicílio. Aqui, vamos guiar e auxiliar no passo a passo do monitoramento da glicemia, um cuidado essencial para a continuidade do tratamento em domicílio. Vamos iniciar apresentando os materiais necessários para o desenvolvimento da verificação da glicemia. O primeiro passo é garantir que você tenha todos os materiais: glicosímetro - aparelho para medir a taxa de glicose (conhecida popularmente como “açúcar”) no sangue; lanceta; tira reagente; algodão; e álcool 70% líquido. Esses materiais serão fornecidos pela equipe de saúde no momento da sua alta hospitalar por meio de sua Unidade Básica de Saúde. Fique tranquilo, você não precisará comprar nada. Importante ressaltar que esse teste de glicemia é necessário ser realizado durante algum tempo, também em domicílio. Ele se faz necessário devido ao órgão estar em recuperação, devido ao uso elevado das doses de imunossupressores e corticoides. Com o passar do tempo e a recuperação do órgão, além dos ajustes na medicação, esse teste não será mais necessário. A equipe de saúde que acompanha você irá lhe informar quando não houver mais a necessidade de realizar o monitoramento da glicose. Atenção: mostrar a higienização da bancada, falar da importância da higienização e, em seguida, mostrar e apontar os materiais em cima da bancada.

Juntamente com a descrição das cenas, os pesquisadores elaboraram a ambiência da cena por meio de desenhos simples, feitos à mão, a fim de demonstrar os cenários em busca de maior aproximação com a realidade vivenciada no domicílio do cliente. Na Figura 1, há um exemplo da explanação das cenas por meio de desenhos simples.

**Figura 1:** Exemplo de um cenário de uma das etapas do vídeo “Cuidados domiciliares relacionados à higiene em domicílio”, Florianópolis, Santa Catarina, Brasil, 2024

A validação de conteúdo do roteiro foi realizada por seis enfermeiros que atuam na prática assistencial, com idade média de 44,5 anos. Entre os participantes, 28,6% possuem quatro anos ou mais de experiência na transição do cuidado/alta hospitalar do transplante hepático; 42,9% possuem mestrado; 51,1% trabalham em instituição pública; e 57,1% desempenham atualmente a função assistencial junto à equipe multiprofissional do transplante hepático.

A Tabela 1 apresenta os principais dados relacionados à validação de conteúdo no que se refere à concordância dos critérios entre os *experts.* Nota-se que a média geral de todos os critérios foi superior a 83%. Os critérios com maior concordância foram a credibilidade, com percentual acima de 89%, seguido do critério concordância com 84,68%. Ainda, destaca-se que não foi pontuado o escore de discordância e discordância parcialmente.

**Tabela 1:** Avaliação dos *experts* (N=06) quanto à validação de conteúdo das cenas do vídeo. Florianópolis, Santa Catarina, Brasil, 2024

Critérios	Concordo (%)	Concordo parcialmente (%)	Discordo parcialmente (%)	Discordo (%)
Objetividade	82,13	17,87	00	00
Simplicidade	80,18	19,82	00	00
Clareza	82,96	17,04	00	00
Credibilidade	89,72	15,27	00	00
Pertinência	84,17	15,85	00	00
Concordância	84,68	15,32	00	00
Média	83,97	16,81	00	00

A seguir, o Quadro 2 apresenta um exemplo do roteiro ajustado pelos pesquisadores após ajustes das recomendações pelos avaliadores. O quadro apresenta a importância da consulta aos *experts,* profissionais da prática, em razão de ser possível observar que houve ajustes importantes e acréscimos fundamentais de informações.

**Quadro 2:** Demonstração do recorte do roteiro elaborado pelos pesquisadores e após sugestão de ajustes por *experts,* Florianópolis, Santa Catarina, Brasil, 2024

Recorte do roteiro definido pelos pesquisadores	Recorte do roteiro após ajustes das sugestões por *experts*
Quanto à não utilização de animais, destaca-se que esses possuem bactérias, vírus e outros micro-organismos que são invisíveis aos nossos olhos. No entanto, qualquer contato do cliente, o qual já está com a imunidade baixa, pode resultar na aquisição dessas bactérias, vírus e outras contaminações que podem estar presentes nos animais.	Quanto à não utilização de animais, destaca-se que há necessidade de minimizar ou evitar contato com animais, como também não manipular as fezes e urina do seu pet. É muito importante, nesta fase, que você evite dormir com o animal de estimação e mantenha todas as vacinas em dia para evitar doenças. Os animais possuem bactérias, vírus e outros micro-organismos que são invisíveis aos nossos olhos; por isso, é necessária a higienização das mãos após brincar. Qualquer contato do cliente, o qual já está com a imunidade baixa, pode resultar na aquisição dessas bactérias, vírus e outras contaminações, que podem estar presentes nos animais.

Após a validação do conteúdo do roteiro pelos enfermeiros, a terceira etapa foi conduzida em relação ao desenvolvimento e produção do vídeo. O vídeo foi produzido, editado e disponibilizado no *YouTube®,* e pode ser acessado por meio dos *links:* https://youtu.be/jQwPgwY54ZY e https://youtu.be/qaI9RxtEIko.

Para as duas últimas etapas, implementação e avaliação, participaram da pesquisa nove clientes, sendo cinco homens e quatro mulheres. A média de idade foi de 44,5 anos, e a média de tempo da transição do cuidado entre hospital e domicílio foi de 31 dias.

A Tabela 2 demonstra os resultados em relação à avaliação do vídeo pelos clientes. A média geral do escore “muito bom” foi superior a 61%, seguido do escore “bom”, com uma média de 23,8%, o que informa uma avaliação positiva diante da tecnologia desenvolvida. Os critérios mais bem avaliados pelo escore “muito bom” foram em relação à contribuição do vídeo no conhecimento dos cuidados domiciliares, às informações sobre as etapas dos cuidados e à perspectiva dessas informações em melhorar os cuidados domiciliares.

**Tabela 2:** Avaliação do vídeo pelos participantes (N = 09 clientes), Florianópolis, Santa Catarina, Brasil, 2024

Critérios	Muito bom (%)	Bom (%)	Razoável (%)	Ruim (%)
1 - Duração do vídeo	42,8	42,8	14,4	00
2 - Figuras, personagens e materiais do vídeo	50,0	33,3	16,7	
3 - Contribui para o aprimoramento do conhecimento quanto aos cuidados domiciliares	71,4	14,3	00	14,3
4 - Informações do vídeo e o passo a passo para realizar o cuidado estão claros	71,4	14,3	00	14,3
5 - Informações podem melhorar os cuidados e apoiar a gestão desses cuidados em domicílio	71,4	14,3	00	14,3
Média	61,4	23,8	6,2	8,6

## DISCUSSÃO

A educação em saúde, por meio de tecnologias, representa uma estratégia valiosa para auxiliar nas dificuldades do pós-operatório, haja vista que os vídeos educativos servem como ferramentas de aprimoramento para os familiares, cuidadores e clientes submetidos ao transplante hepático. Estudos indicam que os clientes apresentam, frequentemente, dificuldades e fragilidades para realizar as técnicas e/ou cuidados necessários que devem ser realizados em domicílio. Ainda, há preocupações com o cuidado e manutenção do ambiente domiciliar adequado para reinserção do cliente no retorno a sua casa após o transplante. Assim, compreende-se a importância de a equipe multiprofissional ter estratégias eficazes para minimizar essas inseguranças ou dúvidas que podem surgir após a alta, tendo em vista que a não compreensão desses cuidados pode acarretar problemas relacionados ao controle ineficaz, gestão deficiente dos cuidados e higienização inadequada. Tais situações aumentam o risco de intercorrências e complicações^(22, 23)^.

Neste estudo, houve uma proposta dos pesquisadores para buscar identificar as necessidades de cuidados desses clientes, com a elaboração do roteiro do vídeo para atender às individualidades e informações essenciais acerca dos cuidados domiciliares. Importante destacar a necessidade da construção de ferramentas que estejam aptas a atender os usuários. Somente assim, essas ferramentas serão utilizadas e poderão alcançar a efetividade desejada aos objetivos propostos para o cuidado^(24, 25, 26)^.

Durante o desenvolvimento do estudo, os pesquisadores voltaram sua atenção para aprimorar as informações do roteiro com apoio de enfermeiros que atuam na transição do cuidado. Essa etapa é considerada importante, uma vez que esses profissionais podem avaliar e ajustar as estratégias de gestão dos cuidados domiciliares com base no dia a dia do atendimento.

Estudos destacam a importância de o produto ser avaliado por profissionais com experiência na área, com a finalidade de agregar de forma prática na ferramenta a ser elaborada, trazendo sugestões e modificações para que o produto ou tecnologia fique acessível ao público-alvo. A avaliação é crucial por várias razões como: validar a qualidade do vídeo, garantindo que ele atenda aos padrões e práticas recomendadas; identificar problemas ou fraquezas; e oferecer alternativas. Além disso, o *feedback* dos especialistas auxilia no refinamento, viabilidade técnica e aprimoramento do vídeo^(27, 28, 29)^.

Outrossim, pontuado por diferentes autores, é relevante a inserção dos usuários em todas as etapas do processo de criação de materiais educativos, visto que esta inclusão permite que a ferramenta seja simples e acessível, de fácil compreensão e manipulação do recurso pelo público-alvo. Essa aproximação da realidade insere a situação diária de cada cliente, favorecendo a disponibilidade de espaço para sanar dúvidas, bem como compreender que tais informações são capazes de empoderar e apoiar a condução dos cuidados domiciliares^(24, 28, 30)^.

Neste estudo, o vídeo foi disponibilizado para clientes, familiares e cuidadores que estavam em fase de adaptação domiciliar, os quais representam momentos que demandam a realização de cuidados e controles domiciliares. Assim, tiveram a possibilidade de acessar essa ferramenta a todo o momento que julgavam necessário. Estudos apontam que, considerando a diversidade dos usuários que utilizam ferramentas de cuidado, essas precisam ser utilizadas por um período distinto, buscando avaliação da compreensão e aceitação desse produto^(31, 32)^.

Destaca-se que a ferramenta deste estudo foi disponibilizada via *YouTube*® como um recurso de procura rápida e facilitada, conforme preconizado no desenvolvimento de métodos de elaboração e criação de materiais educativos. Compreende-se que as Tecnologias da Informação e Comunicação em Saúde (TICS) permitem o armazenamento e processamento de dados digitais, acesso a informações e comunicações à distância. Portanto, o uso das TICS busca auxiliar e otimizar a assistência para tomada de decisões tanto na prática clínica quanto no monitoramento, avaliação das ações e Educação Permanente em Saúde^(33)^.

Na perspectiva do uso e avaliação dos usuários, a participação desse público permite o aprimoramento da ferramenta, tornando-a didática para os futuros usuários. Através da definição de metas e objetivos factíveis da função do vídeo, os usuários utilizaram os vídeos durante um mês, possibilitando pontuar as necessidades de mudanças. Autores apontam que a participação dos usuários enriquece o contexto da ferramenta, além de aproximar-se ao máximo da demanda, com reformulações e ajustes que tornam o produto mais acessível^(29, 34)^.

Outros estudos que produziram vídeos com a participação dos usuários tiveram resultados similares aos deste estudo. Os autores reforçam que essa ferramenta de vídeo educativo permite que os clientes observem na prática os procedimentos realizados a qualquer momento, podendo ter a flexibilidade de pausar e rever diversas vezes, sendo uma estratégia eficaz de promoção da saúde^(14, 15)^. Todavia, tais tecnologias devem ser desenvolvidas com a participação efetiva dos usuários em todas as etapas, visto que, para ter um impacto positivo, deve ser avaliado e ajustado por pessoas que efetivamente possuem vivências na realidade da mudança de rotina imposta pelo transplante, aproximando do contexto vivido e estimulando a representatividade no desenvolvimento de estratégias de cuidado^(16, 17, 35)^.

Por serem ferramentas ilustrativas e interativas, os vídeos educativos possuem boa aceitabilidade pelo público no que concerne à usabilidade e divulgação. Ainda, estimula a oportunidade de melhoria no conhecimento dos usuários após utilização do vídeo^(25, 36)^, gerando impacto na qualidade de vida do transplantado.

Considerando tais dados apresentados, bem como os resultados obtidos em outros estudos citados anteriormente, percebe-se que a aplicação da ferramenta deste estudo no cotidiano de orientações para a alta hospitalar pode trazer benefícios importantes, seja para clientes que irão usufruir deste vídeo em todos os momentos que desejarem, seja para a equipe multiprofissional, que terá mais essa estratégia de cuidado como instrumento na adesão ao tratamento junto aos clientes submetidos ao transplante hepático.

### Limitações do estudo

As limitações deste estudo estão relacionadas a dificuldades de identificar profissionais enfermeiros com *expertise* na transição do cuidado entre hospital e domicílio com foco no transplante hepático. Além disso, o vídeo educativo foi validado por clientes que realizaram transplante hepático em uma única instituição.

### Contribuições para a área da enfermagem

Este estudo proporciona aprimoramento da gestão do cuidado no domicílio em razão do cliente que realizou o transplante hepático, juntamente com seu familiar e/ou cuidador, poder acessar a qualquer momento as informações relacionadas aos cuidados domiciliares. Assim, há possibilidade de fortalecer a educação em saúde e gerenciamento dos cuidados, além de apoiar a equipe multiprofissional do transplante através de ferramenta educacional, melhorando adesão ao tratamento e trazendo autonomia aos clientes. Os resultados da validação entre profissionais *experts* e clientes demonstram a viabilidade de incluir a ferramenta educacional na rotina de preparo para alta hospitalar do cliente transplantado hepático, contribuindo para a transição entre hospital e domicílio.

## CONSIDERAÇÕES FINAIS

O estudo desenvolveu e validou um vídeo para promover educação em saúde de clientes submetidos ao transplante hepático. A referida ferramenta educacional foi formada por cenas em que é apresentado o passo a passo dos cuidados diários a ser realizado no domicílio voltado a: higiene oral; monitoramento da glicemia; cuidados com a ferida operatória; aplicação da insulina; controles diários (verificação dos sinais vitais; mensuração da diurese e peso); e desenvolvimento dos controles diários e sinais de alerta para intercorrências e complicações. Ainda, está formado por cenas relacionadas aos cuidados com o ambiente domiciliar e cuidados com higiene pessoal, como organização e limpeza do ambiente, cuidados com o uso da máscara, e visitas domiciliares.

Ainda, ressalta-se que esse vídeo foi implementado junto aos clientes submetidos ao transplante hepático, avaliando se essa ferramenta atende às suas necessidades em domicílio.

A concordância entre a avaliação dos profissionais com *expertise,* bem como pelos clientes, demonstra que as avaliações foram consideradas boas, o que indica que o vídeo educacional está com o seu conteúdo validado. Ainda, tal ferramenta poderá ser utilizada pelos clientes, bem como pela equipe multiprofissional na transição do cuidado entre hospital e domicílio do cliente transplantado hepático.

## Data Availability

https://doi.org/10.17605/OSF.IO/JCFMP
